# Reconstruction of nasal airway in patients of class IIId maxillary defect with vastus lateralis muscle-chimeric double skin paddle ALT flap

**DOI:** 10.1186/s12903-022-02635-y

**Published:** 2022-12-08

**Authors:** Shao-Yun Hsu, Shane D. Morrison, Mehmet Emin Cem Yildirim, Pin-Keng Shih

**Affiliations:** 1 Division of Reconstructive Microsurgery, Department of Plastic and Reconstructive Surgery, College of Medicine, Chang Gung Memorial Hospital, Chang Gung University, Taoyuan, Taiwan; 2 Division of Plastic Surgery, Department of Surgery, University of Washington Medical Center, Seattle, WA USA; 3 Department of Plastic Reconstructive and Aesthetic Surgery, Florence Nightingale Hospital, Demiroglu Science University, Istanbul, Turkey; 4 School of Medicine, China Medical University, Taichung, Taiwan; 5 Department of Surgery, China Medical University Hospital, Taichung, Taiwan

**Keywords:** Class IIId maxillary defect, Vastus lateralis muscle-chimeric double skin paddle ALT flap, Mouth breathing

## Abstract

**Background:**

A reconstructive challenge in patients with class IIId maxillary defect is how to obliterate the defect and restore a patent nasal airway. The current strategy using the single anterolateral thigh (ALT) fasciocutaneous flap for reconstruction may result in permanent mouth breathing. As the ALT flap was a common option in reconstruction processes, this study aimed to evaluate the benefits of vastus lateralis (VL) muscle-chimeric double skin paddle ALT flap in simultaneous defect and nasal airway reconstruction.

**Methods:**

This study included 21 patients with class IIId maxillary defect who underwent free ALT flap reconstruction (n = 11, single ALT flap group; n = 10, VL muscle-chimeric double skin paddle ALT flap (chimeric ALT flap) group) at the China Medical University Hospital from August 2015 to September 2019. Associated parameters collected for analysis included gender, age, body mass index (BMI), operative time, hospitalization, clinical stage, preoperative treatment, flap/defect size, comorbidities, postoperative RT, mouth breathing and short/long term complications.

**Results:**

No significant differences were observed in age, BMI, hospitalization, clinical stage, preoperative treatment, defect size, comorbidities, and postoperative RT between the two groups; however, the chimeric ALT flap group as dominated by male patients (p = 0.009), and had longer operative times (12.1 h vs. 10.1 h, p = 0.002) and larger flap sizes (180 cm^2^ vs. 96.7 cm^2^, p = 0.013). Compared with the chimeric ALT flap group, the single ALT flap group suffered from permanent mouth breathing.

**Conclusion:**

Nasal airway reconstruction should be considered in patients with class IIId maxillary defect. Compared to the single ALT flap, the chimeric ALT flap is a superior reconstructive option for patients with class IIId maxillary defect, although a longer surgical duration and larger flap size are required.

## Introduction

An adequate head and neck reconstruction requires defect coverage and functional restoration. Although single microsurgical flaps provide abundant volume and reliable defect coverage perfusion, their bulkiness and inflexibility may compress the pedicle, digestive tract, and respiratory tract [1]. Conversely, double microsurgical flaps waive the above mentioned issues; however, they are associated with tedious work, increased risk of reanastomosis, and donor site complications due to multiple flap harvests and transfers [2].

Microsurgical chimeric flaps possess major advantages while serving as both single and double flaps. Each unique part comprising the chimeric flap restores the corresponding functional [3] and aesthetic [4] defects via a single microsurgical procedure. Liu et al. proposed the double-paddle peroneal chimeric flap for oral commissuroplasty and lip defect reconstruction of through-through cheek defects [5] Ooi ASH et al. practice the chimeric medial sural artery perforator flap for simultaneous tongue and mouth floor defect reconstruction [6]. These findings imply that the independent components of a chimeric flap may benefit the respective reconstructive results as double microsurgical flaps but reduce their labor and risk of multiple anastomoses.

Current reconstruction strategies for class III maxillary defects mainly focus on repairing the orbit floor and providing facial skin support, adequate soft tissue [7], or strong bone framework support [8]. In contrast, nasal airway reconstruction was considered less important because most oncological resections involve the unilateral nasal airways (class IIIb maxillary defect). Patients with class IIId maxillary defects may require simultaneous filling and airway reconstruction, which is not achieved in most single free flaps [9]. Therefore, the chimeric or double flaps might be the solution to this challenge.

The vastus lateralis (VL) muscle-chimeric double skin paddle anterolateral thigh (ALT) flap has been widely applied in heel [10], head/neck [11], and upper extremity [12] defect reconstruction. At present, it is less well known how to simultaneously solve maxillary defect and re-establish a patent nasal airway in patients with class IIId maxillary defect. This study aimed to survey the importance of a nasal airway in class IIId maxillary defect and evaluate the benefits of the VL muscle-chimeric double skin paddle ALT flap in overcoming current challenges.

## Material methods

### Patient data

Medical records of patients with class IIId maxillary defect (Brown classification [9]), who underwent free ALT fasciocutaneous flaps with VL muscle at the China Medical University Hospital from August 2015 to September 2019, were retrospectively reviewed. This study was approved by the research ethics committee of the China Medical University and Hospital (CMUH107-REC3-047). Patients were divided into two groups: group 1 comprised of patients who used a single ALT fasciocutaneous flap with VL muscle (single ALT flap group (n = 11)) and group 2 comprised of patients who used a VL muscle-chimeric double skin paddle ALT flap (chimeric ALT flap) group (n = 10). Microsurgical double skin paddle chimeric ALT flap transfers were conducted if > 2 sizable perforators supplied the ALT flap. At post-discharge, all patients were followed up for a period of one month and every six months thereafter. All participants provided informed consent prior to participation.

Demographic data, including gender, age, body mass index (BMI), operative time, hospitalization, clinical stage, preoperative treatment, flap/defect size, comorbidities, postoperative radiotherapy (RT), mouth breathing and short/long term complications were recorded and compared.

### Statistical analysis

The parameters were not normally distributed. Due to varying sample sizes between the two groups, the nonparametric Mann–Whitney U-test was used to determine the magnitudes of between-group differences. Meanwhile, the Fisher exact test was used to determine differences between the two groups based on postoperative complications. *P*-values of < 0.05 were considered statistically significant. The GraphPad for Windows version 5.01 was used for all statistical analyses.

### Chimeric ALT flap harvesting

**Fig. 1:**
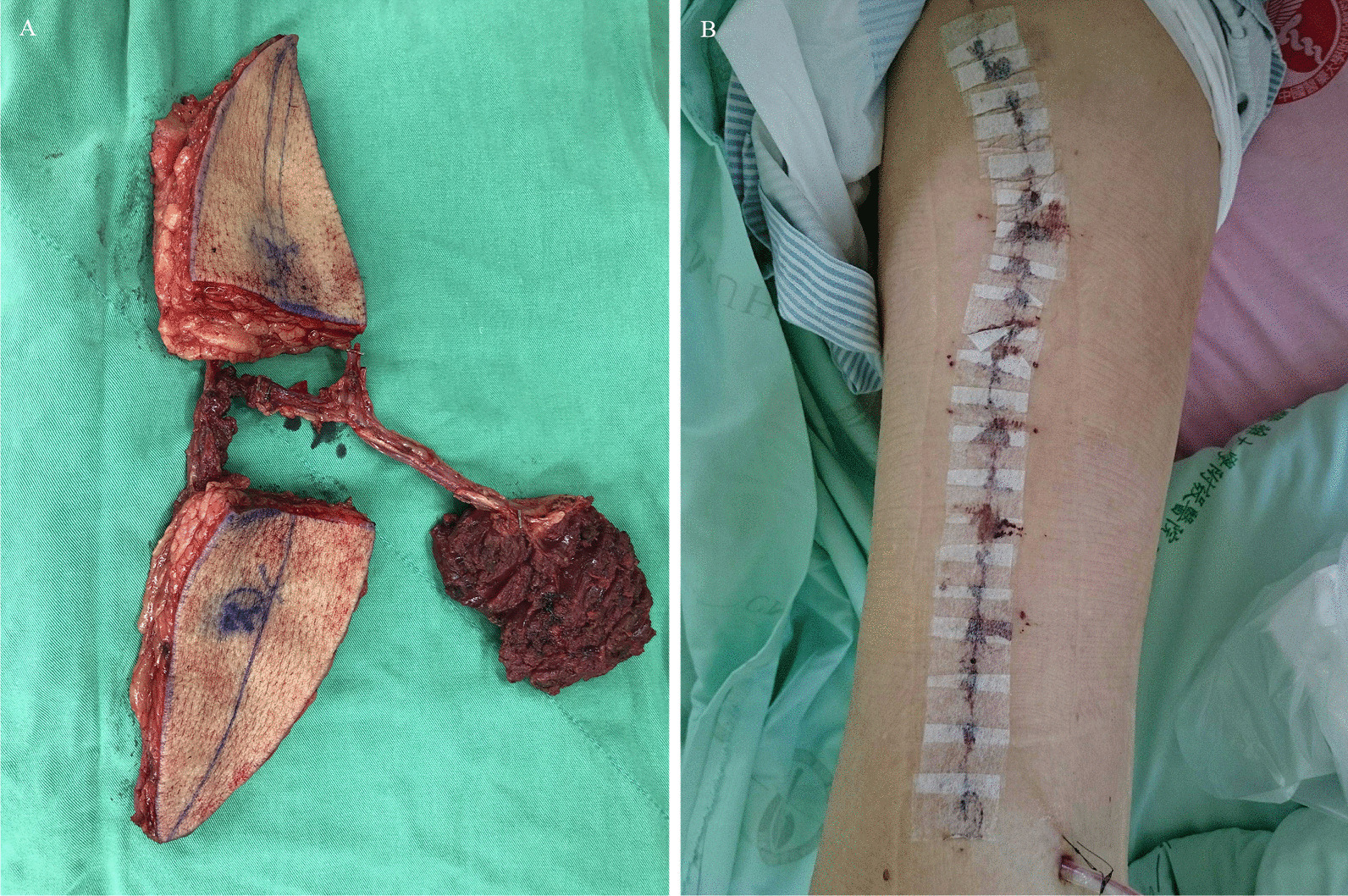
Representative image of the VL muscle-chimeric double skin paddle ALT flap (chimeric ALT flap) (**A**). An immediately postoperative image of the flap donor site (**B**)

Perforators to the skin paddle were marked using handheld Doppler ultrasonography before skin incision. A medial incision was made along the rectus femoris (RF) septum, which then deepened into the subfascial layer. The dissection was performed in the medial to lateral direction. The intramuscular perforators and pedicles between the RF and VL muscles were cautiously isolated. The oblique and descending branch of the lateral circumflex femoral artery were designed for skin paddle and VL muscle supply, respectively. The skin paddles were split when at least two sizable perforators were found to bifurcate from a single pedicle (Fig. 1A). The ALT flap was harvested and transposed to the defect. The donor site was closed layer-by-layer using Vicryl 2−0, 3−0 and Monocryl 4−0 sutures (Fig. 1B). Finally, the Jackson–Pratt drain was routinely placed underneath the subcutaneous layer.

## Results

The single ALT group consisted of two male and nine female patients with a mean age of 64 years, BMI of 21.9 kg/m^2^, operative time of 10.1 h, and length of hospitalization stay of 14 days. The mean defect and flap sizes were 30.3 and 96.7 cm^2^, respectively.

The chimeric ALT flap group comprised eight male and two female patients with a mean age of 66.2 years, BMI of 20.7 kg/m^2^, operative time of 12.1 h, and length of hospitalization stay of 14.8 days. The mean defect and flap sizes were 29.7 and 180 cm^2^, respectively.

No significant differences were observed in age, BMI, length of hospitalization stay, clinical stage, preoperative treatment, defect size, comorbidities, and postoperative RT between the two groups. Compared with the single ALT flap group, the chimeric ALT flap group was dominated by male patients (*p* = 0.009), and had longer operative times (12.1 h vs. 10.1 h, *p* = 0.002) and larger flap size (180 cm^2^ vs. 96.7 cm^2^, *p* = 0.013) (Table 1).

**Table 1:** Basic characteristics of patients

	Single ALT flap (n = 11)	Chimeric ALT flap (n = 10)	p- value
Gender (M/F)	2/9	8/2	0.009*
Age	64.0	66.2	0.721
BMI (kg/m^2^)	21.9	20.7	0.18
Operative time (h)	10.1	12.1	0.002*
Hospitalization (days)	14.0	14.8	0.805
Clinical stage IV Recurrent	8 3	10 0	0.214 0.214
Preoperative treatment			
CT	2	1	1.000
RT	3	1	0.587
Flap donor site managements	Primary closure	Primary closure	–
Defect size (cm^2^)	30.3	29.7	0.707
Flap size (cm^2^)	96.7	180	0.013*
Comorbidities			
Hypertension	5	8	0.183
Diabetes mellitus	3	3	1.000
Betel nut	4	6	0.396
Smoking	7	8	0.635
Alcohol	7	7	1.000
Chronic kidney disease	2	1	1.000
Postoperative RT (n)	10	10	1.000

*CT* chemotherapy, *RT* radiotherapy

*p < 0.05.

None of the patients in both groups presented postoperative flap complications. All patients in the single ALT flap group had mouth breathing after tracheostomy closure at a 1-month follow-up. In the long-term follow-up, the single ALT flap group had 11 (100%) and 3 (27%) patients who underwent mouth breathing and periorbital relapse defect repair, respectively. In contrast, the chimeric ALT flap group comprised five (50%) and two (20%) patients who underwent nasal airway dilation and periorbital relapse defect repair, respectively. Patients with single ALT flap reconstruction had significantly short- and long-term mouth breathing than the chimeric ALT flap group (Table 2).

**Table 2:** Summaries of complications in each group

	Single ALT flap (n = 11)	Chimeric ALT flap (n = 10)	p-value
Short-term complications (< 6 months)			
Flap donor site	0	0	–
Recipient site	0	0	–
Mouth breathing	11	0	< 0.0001*
Long-term complications (7–24 months)			
Mouth breathing	11	0	< 0.0001*
Nasal airway dilation	–	5	–
Orbit rim implant exposure	3	2	1.000

*p < 0.05

### Case report

#### Case 1 single ALT flap reconstruction

**Fig. 2:**
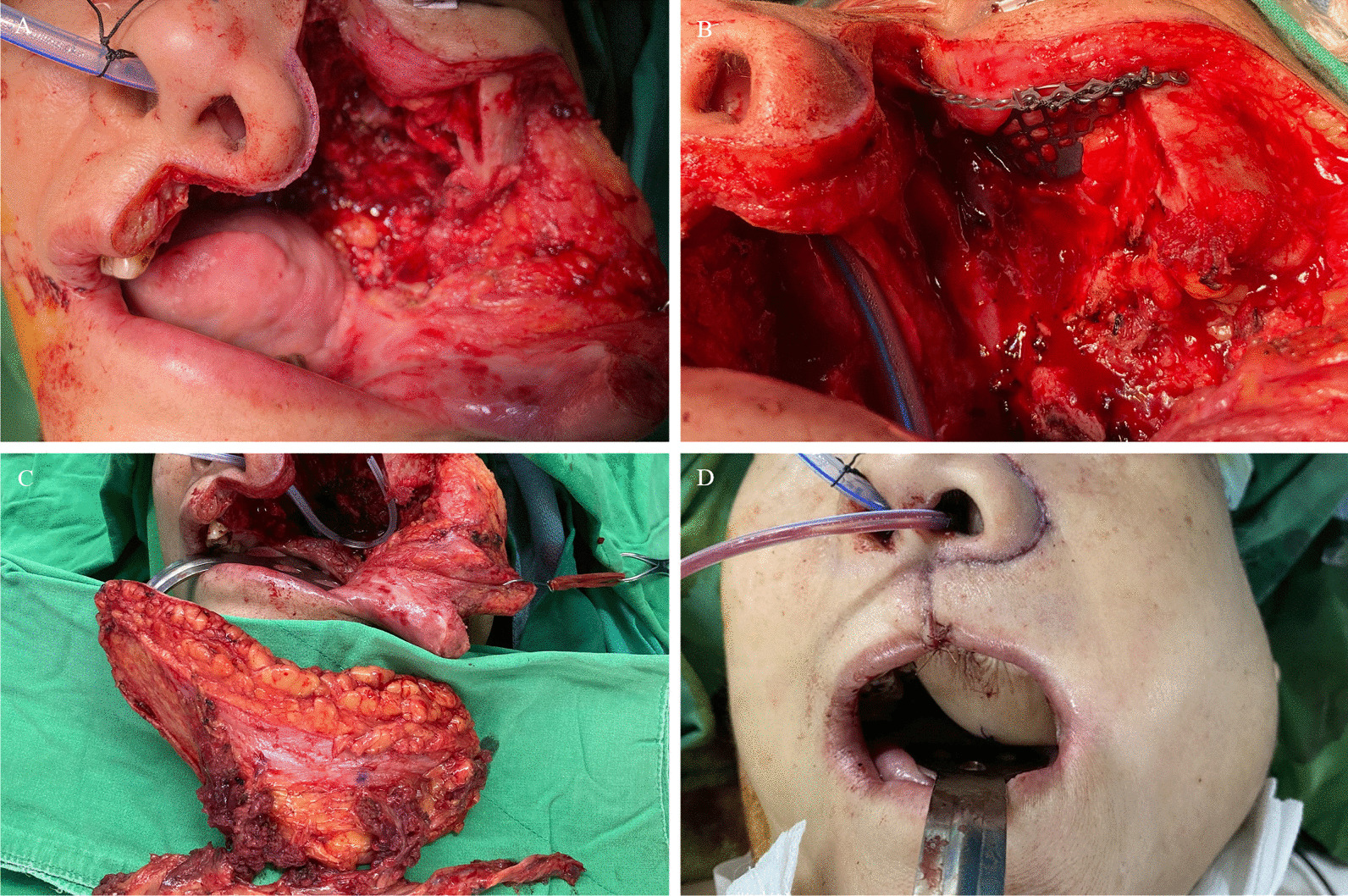
Class IIId maxillary defect noted after wide excision of a left maxilla squamous cell carcinoma (T4N0M0) (**A**). The orbit floor and rim were repaired with a Titanium mesh and C-shape microplate, respectively (**B**). A single ALT fasciocutaneous flap (18 cm × 6 cm) was transposed to the defect (**C**). The VL muscle was used to fill the left maxillary defect, and the skin paddle area was modified in accordance with the defect size (**D**). An immediately postoperative image is shown

A 56-year-old female patient with maxilla squamous cell carcinoma (T4N0M0) underwent bilateral maxillectomy, left orbit floor removal, wide palatal excision, and neck dissection (Fig. 2A). The orbit floor and rim were repaired with a titanium mesh and a C-shape microplate, respectively (Fig. 2B). A free ALT myocutaneous flap (18 cm × 6 cm) was harvested and transposed to the defect (Fig. 2C). The VL muscle was used to fill the left maxillary defect, and the skin paddle area was modified in accordance with the defect size. The pedicle vessel was anastomosed to the left superior thyroid artery (STA) and external jugular vein. An immediately postoperative image is shown in Fig. 2D.

### Case 2 chimeric ALT flap reconstruction

**Fig. 3:**
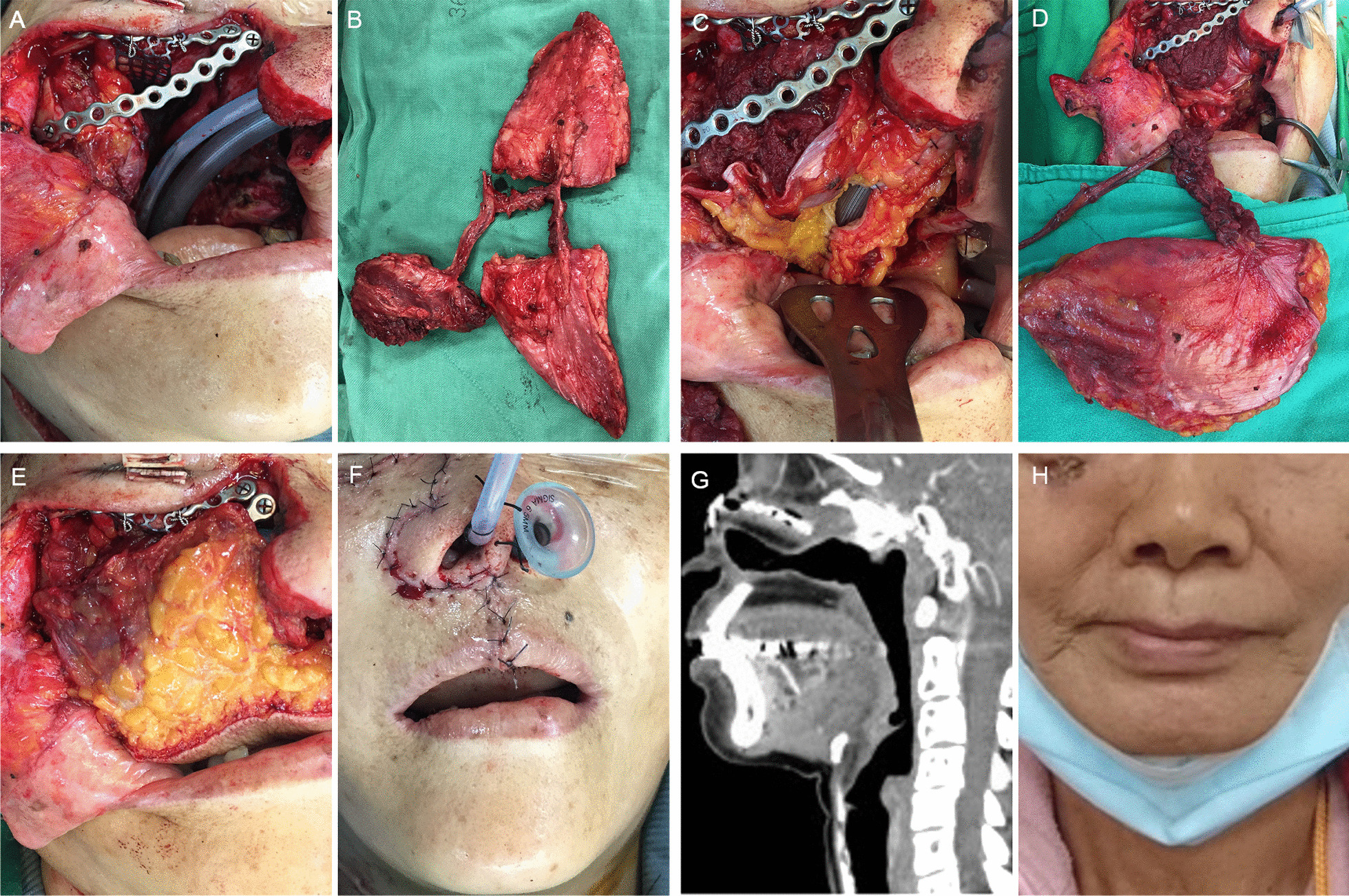
A 45-year-old female patient with maxilla squamous cell carcinoma (T4N0M0) underwent a wide excision and reconstruction of mesh and mini-plates (**A**). The chimeric ALT flap (30 cm × 6 cm) was designed for simultaneous nasal airway and palatal defect reconstruction (**B**). A nasal airway tubing surrounded by a skin paddle was used to guide airway reconstruction (**C**). The VL muscle was transferred to the right maxillary defect (**D**), and the palatal defect was covered with the other skin paddle in accordance with the defect size (**E**). An immediately postoperative image is shown (**F**). At 2-years follow-up, a computed tomography scan revealed partial nasal airway stenosis without compromising the respiratory function (**G**). Postoperative 2-years follow-up image (**H**)

A 45-year-old female patient with maxilla squamous cell carcinoma (T4N0M0) underwent bilateral maxillectomy, right orbit floor removal, wide palatal excision, and neck dissection. The orbit floor and maxillary sinus were reconstructed using mesh and mini-plates (Fig. 3A). A chimeric ALT flap (30 cm × 6 cm) was harvested for simultaneous nasal airway and palatal reconstruction (Fig. 3B). The skin paddle was separated into two parts according to the perforator position and whether each paddle size was adequate for the palatal defect covering and nasal airway wrapping, respectively. The skin paddle designed for tubing airway formation was inset firstly, and the skin paddle-inner nostril mucosa closure was performed with Vicryl 3−0 suture (i.e. posterior wall of nasal airway repaired firstly). Then, a nasal airway tube (removed before hospital discharge) was inserted into the respiratory tract as a guide, and the remaining skin paddle was wrapped to form a nasal airway (Fig. 3C). Then, the descending branch supplying the VL muscle was transferred to the right maxillary defect (Fig. 3D). Finally, the palatal defect was covered with the other skin paddle in accordance with the defect size. (Fig. 3E). The pedicle vessels were anastomosed to the STA and internal jugular vein branch. The subcutaneous layer and skin were sutured using Vicryl 4−0 and Nylon 5−0 sutures (Fig. 3F). In the 2-year follow-up, partial nasal airway stenosis (but without compromising respiratory function) was found on a computed tomography scan (Fig. 3G). The 2-year follow-up image is shown in Fig. 3H.

## Discussions

The respiratory function of patients with class IIId maxillary defect is related to their primary reconstruction. Contrary to the chimeric ALT flap group, this study’s long-term follow-up revealed that all patients in the single ALT flap group suffered from permanent mouth breathing. This finding may imply that normal breathing cannot be achieved in scenarios where the reconstruction plan only involves wound coverage and dead-space obliterations with a non-chimeric bulky flap. Mouth breathing was less reported in other maxillary defect reconstructions with free soft-tissue flap, possibly due to most cases being class IIIb maxillary defects and because the other nasal airway was well-preserved [13]. In addition, compared with the free soft-tissue flap, the free bone flap was still the main treatment option used in class III maxillary defects [14]. Accordingly, the experiences of the authors of this work reflect the importance of nasal airway reconstruction and the superiority of chimeric ALT flap in patients with class IIId maxillary defect.

Mouth breathing impairs those patients’ the quality of life of such patients due to difficulties in maintaining oral hygiene and oral mucosa dryness [15]. A single respiratory tract (instead of having patent oral and nasal airways) increases the risk of choking, shortness of breath, hypoxia, hypercapnia, or even apnea, especially while eating or sleeping [16]. Mouth breathing has also been associated with other class IIId maxillary defects reconstructed with free bone flap. Joseph et al. [17] and Bianchi et al. [8] successfully achieved wound coverage and aesthetic face contouring by free fibular and iliac crest flap, respectively. Because no functional breathing was re-established, most of their patients experienced all the abovementioned disadvantages and risks.

Several challenges were noted during the simultaneous nasal airway reconstruction with the chimeric ALT flap. First, the ALT flap must be carefully designed to be transferred as a chimeric pattern. Having at least three reliable perforators is a prerequisite to providing adequate perfusion of at least two skin paddles and one muscle part. However, this physiological prerequisite notably increases the surgeon’s technical demands, rendering the surgery challenging. Second, bulky skin paddles or a skin paddle with perforators embedded in a muscle trunk are less pliable, making them challenging to be tubed as a nasal airway and to contour the palate simultaneously. On the contrary, these challenges might be overcome by the independent components in a chimeric ALT flap. Most female patients in this study did not achieve the abovementioned physiological prerequisites because females usually have smaller perforators and more bulky skin paddles than males. Therefore, most of the female patients opted for chimeric ALT flap, but underwent single ALT flap reconstructions.

In the current series, most patients who underwent nasal airway reconstructions with chimeric ALT flap are noted to have normal breathing for > 2 years. Still, some of them developed respiratory tract stenosis after receiving adjuvant radiotherapy. The high percentage of adjuvant radiotherapy in the series (Table 1) might be the major cause that led to skin paddle fibrosis and subsequent airway obstruction. Yet, further studies are required for investigating the detailed mechanism.

Post-radiation stenosis could easily be solved using non-surgical dilatation method, whereas an emerging challenge was to alleviate respiratory tract stenosis after long-term follow-up. Balasubramanian et al. used a microsurgical forearm flap with titanium meshes for partial trachea defect reconstruction [18]. Moreover, an animal model trial without clinical application tried to use expanded polytetrafluoroethylene (ePTFE) as nasal airway framework to prevent airway collapse [19]. In the current series, owing to the fact that most patients who underwent bilateral maxillectomy required adjuvant radiotherapy, which may increase the risks of implant exposures; therefore, the authors of this work prefer tubing skin paddle with autologous tissue framework (like rib graft) as the further nasal airway reconstruction option.

Although the chimeric ALT flap provided both nasal airway and palatal defect reconstruction, the reconstructed nasal airway was still fragile and with an increased risk of collapse after completing postoperative radiotherapy. Regarding the issue of mouth breathing after long-term follow-up, this preliminary result suggests that patients with chimeric ALT flap reconstruction could have, at least, a normal breath type of 2-year duration even there were half patients received dilation treatment. However, owing to its retrospective nature and limited case number, further studies are warranted.

To our knowledge, this study is the first to use chimeric ALT flap to reconstruct the nasal respiratory tract, obligate maxillary, and palate defects simultaneously. In fact, mouth breathing was a common challenge in class IIId maxillary defect reconstruction regardless of whether free bony (free fibular or tensor-fascia-lata with iliac crest) or soft-tissue flaps (free rectus abdominis, deep circumflex iliac artery flap, radial forearm flap, or ALT flap) were used [17]. The patent nasal airway should be a major functional consideration in class IIId maxillary defect reconstruction. These experiences reflect the importance of nasal airway reconstruction and the superiority of chimeric ALT flap in defect reconstruction.

The current study had several limitations. First, the number of participants was relatively small, and the study was performed at a single facility. Hence, these results might not be applicable to all institutions. Second, the texture and shape of chimeric ALT flap changes with radiotherapies, and this study might not have had an adequate follow-up period. Therefore, further studies with a more extended follow-up period should be conducted. Finally, biases might have existed due to the study’s retrospective nature.

## Conclusion

Nasal airway reconstruction should be considered in patients with class IIId maxillary defect. Compared to the single ALT flap, the chimeric ALT flap is a superior reconstructive option for patients with class IIId maxillary defect, although a longer surgical duration and larger flap size are required.

## Data Availability

The datasets used and/or analyzed during the current study are available from the corresponding author on reasonable request.

## Abbreviations

BMI: Body mass index
RF: Rectus femoris
STA: Superior thyroid artery
VL: Vastus lateralis
